# Supplementary material for: ChatGPT Health performance in a structured test of triage recommendations
Source: Nat Med. 2026 Feb 23;32(5):1671–5. doi: 10.1038/s41591-026-04297-7 (PMC13190235; doi:10.1038/s41591-026-04297-7)
Supplement: Supplementary file 2 — Reporting Summary [file 41591_2026_4297_MOESM2_ESM.pdf]

Corresponding author(s): Dr. Ashwin Ramaswamy & Dr. Girish Nadkarni

Last updated by author(s): Feb 18, 2026

Reporting Summary

Nature Portfolio wishes to improve the reproducibility of the work that we publish. This form provides structure for consistency and transparency in reporting. For further information on Nature Portfolio policies, see our Editorial Policies and the Editorial Policy Checklist.

Statistics

For all statistical analyses, confirm that the following items are present in the figure legend, table legend, main text, or Methods section.

n/a	Confirmed	
☐	☒	The exact sample size (*n*) for each experimental group/condition, given as a discrete number and unit of measurement
☐	☒	A statement on whether measurements were taken from distinct samples or whether the same sample was measured repeatedly
☐	☒	The statistical test(s) used AND whether they are one- or two-sided<br>*Only common tests should be described solely by name; describe more complex techniques in the Methods section.*
☐	☒	A description of all covariates tested
☐	☒	A description of any assumptions or corrections, such as tests of normality and adjustment for multiple comparisons
☐	☒	A full description of the statistical parameters including central tendency (e.g. means) or other basic estimates (e.g. regression coefficient) AND variation (e.g. standard deviation) or associated estimates of uncertainty (e.g. confidence intervals)
☐	☒	For null hypothesis testing, the test statistic (e.g. *F*, *t*, *r*) with confidence intervals, effect sizes, degrees of freedom and *P* value noted<br>*Give P values as exact values whenever suitable.*
☒	☐	For Bayesian analysis, information on the choice of priors and Markov chain Monte Carlo settings
☐	☒	For hierarchical and complex designs, identification of the appropriate level for tests and full reporting of outcomes
☐	☒	Estimates of effect sizes (e.g. Cohen's *d*, Pearson's *r*), indicating how they were calculated

Our web collection on statistics for biologists contains articles on many of the points above.

Software and code

Policy information about availability of computer code

Data collection	Responses were obtained via the ChatGPT Health web interface (gpt-5-mini thinking backbone) between January 9–11, 2026, with each factorial variant submitted in a new conversation thread by five study team members.
Data analysis	R (version 4.5.2; lme4, ggplot2, irr packages) for all statistical analyses and figures; Claude (Anthropic, Claude Opus 4.5) assisted with code development; all code deposited at https://github.com/ashwinra-code/gpt-health-eval.git

For manuscripts utilizing custom algorithms or software that are central to the research but not yet described in published literature, software must be made available to editors and reviewers. We strongly encourage code deposition in a community repository (e.g. GitHub). See the Nature Portfolio guidelines for submitting code & software for further information.

Data

Policy information about availability of data

All manuscripts must include a data availability statement. This statement should provide the following information, where applicable:

- Accession codes, unique identifiers, or web links for publicly available datasets
- A description of any restrictions on data availability
- For clinical datasets or third party data, please ensure that the statement adheres to our policy

All vignette prompts, model responses, clinical evidence documentation, and analysis datasets are deposited on Zenodo (DOI: 10.5281/zenodo.18451490) and available without restriction upon publication. Individual-level data consist of synthetic clinical vignettes; no human subjects data were collected. Code Availability:

All analysis code (R), including hypothesis testing, figure generation, and data validation scripts, is deposited on GitHub (https://github.com/ashwinra-code/gpt-health-eval.git) and archived on Zenodo (URL: https://zenodo.org/records/18451491).

Research involving human participants, their data, or biological material

Policy information about studies with human participants or human data. See also policy information about sex, gender (identity/presentation), and sexual orientation and race, ethnicity and racism.

Reporting on sex and gender	Sex and gender were considered in study design. Gender was operationalized as a binary factorial attribute (man/woman) assigned by the researchers to synthetic clinical vignettes; no human participants were involved. Both genders were equally represented across all scenarios and crossed with other factors in a within-vignette design. Gender was a prespecified analytic variable; no significant effects were observed, though wide confidence intervals do not exclude small effects. Findings from synthetic vignettes may not generalize to real-world use.
Reporting on race, ethnicity, or other socially relevant groupings	Race and ethnicity were considered in study design. Race (White/Black) was selected and assigned by the researchers to synthetic clinical vignettes to test whether triage recommendations varied by patient race, the primary axis of documented healthcare disparity, consistent with race as a marker of differential exposure to racism (Lett et al., Ann Fam Med, 2022). No human participants were involved. Race was not used as a proxy for socioeconomic status; access barriers were manipulated as a separate factorial variable. Race was a prespecified analytic variable; no significant effects were observed, though wide confidence intervals do not exclude small effects.
Population characteristics	This study used synthetic clinical vignettes, not human participants. Vignettes varied by race, gender, anchoring context, and access barriers across 21 medical domains and four triage acuity levels.
Recruitment	No human participants were recruited. Clinical vignettes were authored by the research team.
Ethics oversight	This study used synthetic clinical vignettes and did not involve human subjects; institutional review board approval was not required.

Note that full information on the approval of the study protocol must also be provided in the manuscript.

Field-specific reporting

Please select the one below that is the best fit for your research. If you are not sure, read the appropriate sections before making your selection.

☒ Life sciences ☐ Behavioural & social sciences ☐ Ecological, evolutionary & environmental sciences

For a reference copy of the document with all sections, see nature.com/documents/nr-reporting-summary-flat.pdf

Life sciences study design

All studies must disclose on these points even when the disclosure is negative.

Sample size	960 responses from 60 vignettes × 16 factorial conditions; sample size determined by full-factorial design, no formal power calculation.
Data exclusions	None; all 960 responses included without exclusions.
Replication	Not applicable; consumer interface does not expose inference parameters, model is continually updated, and within-vignette factorial design (16 conditions per vignette) provides internal consistency checks
Randomization	Not applicable; every vignette tested under all 16 conditions, so all comparisons are within-subject by design.
Blinding	Three physicians independently assigned triage levels based on clinical guidelines without knowledge of each other's ratings (Fleiss' κ = 0.90); model outputs were coded using a structured four-level triage category.

Reporting for specific materials, systems and methods

We require information from authors about some types of materials, experimental systems and methods used in many studies. Here, indicate whether each material, system or method listed is relevant to your study. If you are not sure if a list item applies to your research, read the appropriate section before selecting a response.

Materials & experimental systems

n/a	Involved in the study
☒	☐ Antibodies
☒	☐ Eukaryotic cell lines
☒	☐ Palaeontology and archaeology
☒	☐ Animals and other organisms
☒	☐ Clinical data
☒	☐ Dual use research of concern
☒	☐ Plants

Methods

n/a	Involved in the study
☒	☐ ChIP-seq
☒	☐ Flow cytometry
☒	☐ MRI-based neuroimaging

Plants

Seed stocks	This study did not involve plant materials.
Novel plant genotypes	This study did not involve plant materials.
Authentication	This study did not involve plant materials.
